# Are Platelet Volume, Density, and the Homeostatic Blueprint the Missing Link in Platelet-Rich Plasma Therapies? An Evidence-Based Narrative Review

**DOI:** 10.3390/jcm15187126

**Published:** 2026-09-14

**Authors:** Peter A. Everts, Luga Podesta, Jane Fitzpatrick, Leonardo P. Oliveira, José Fábio Lana, André van Zundert, Mikel Sánchez, Diego Delgado, Christine Brown Mahoney, Glenn Flanagan, Robert Alexander, Paul Harrison

**Affiliations:** 1 Medical School (GBCS), The University of Queensland, Brisbane, QLD 4006, Australia; a.vanzundert@uq.edu.au; 2 Medical School, Max Planck University Center (UniMAX), Indaiatuba 13343-060, SP, Brazil; josefabiolana@gmail.com; 3 Regenerative Medicine, Orthoregen International Course, Indaiatuba 13334-170, SP, Brazil; 4 Bluetail Medical Group and Podesta Orthopedic Sports Medicine, Naples, FL 34109, USA; lugamd@aol.com; 5 College of Osteopathic Medicine, Orlando, FL 34787, USA; 6 Faculty of Medicine, Dentistry and Surgery, University of Melbourne, Parkville, VIC 3010, Australia; jane.fitzpatrick@unimelb.edu.au; 7 Levitetz Department of Orthopedic Surgery, Cleveland Clinic Foundation, Weston, FL 33331, USA; oliveil@ccf.org; 8 Clinical Research, Anna Vitória Lana Institute (IAVL), Indaiatuba 13334-170, SP, Brazil; 9 Mayne Academy of Critical Care at The University of Queensland, Brisbane, QLD 4006, Australia; 10 Arthroscopic Surgery Unit and Advanced Biological Therapy Unit, Hospital Vithas Vitoria, 01008 Vitoria-Gasteiz, Spain; diego.delgado@hospitalmiks.com; 11 Bio-Statistics College of Business, Minnesota State University, Mankato, MN 56001, USA; cbmahoney2013@charter.net; 12 Naples Regenerative Institute, Naples, FL 34103, USA; gdoc33@gmail.com; 13 Regenevita Biocellular Aesthetic and Reconstructive Surgery, Wound Healing, Hamilton, MT 59840, USA; rwamd1914@gmail.com; 14 Department of Surgery and Maxillofacial Surgery, University of Washington, Seattle, WA 988104, USA; 15 Department of Inflammation and Aging, School of Infection, Inflammation and Immunology, College of Medicine and Health, University of Birmingham, Birmingham B15 2TT, UK; p.harrison.1@bham.ac.uk

**Keywords:** platelet-rich plasma, orthobiology, homeostasis, platelets, platelet density, mean platelet volume, platelet distribution width, heterogeneity, centrifugation, bioformulation

## Abstract

**Background/Objectives:** Platelet-rich plasma (PRP) has been extensively used in clinical practice to treat musculoskeletal (MSK) and spinal maladies. PRP applications and preparation methods focus on variables such as delivery technique, formulation, and device performance. Few PRP preparation methods consider the variability in the biological status of the individual patient following a comprehensive homeostatic tissue repair response. In recent years, more information has become available for addressing the efficacy of PRP devices and preparation methods. Furthermore, the impact of the PRP dose and bioformulation on different pathoanatomical tissues and patient outcomes has been more widely discussed. This review aims to examine different variables in the optimization of PRP preparation methods, focusing on several platelet indices that influence hematopoiesis, subsequent to homeostasis and neuronal regulation, and how this relates to the release of platelets with variable densities, the mean platelet volume (MPV), platelet distribution width (PDW), and ultimately platelet concentration. **Methods:** A literature review was conducted using the PubMed, Medline, and Cochrane databases. **Results:** The literature indicates that the overall physiological platelet dynamics, from bone marrow megakaryocytes to peripheral blood release, differ continuously between health and disease. This study demonstrates that variations in platelet density, as indicated by mean platelet volume (MPV), and platelet count are associated with deviations in the total platelet distribution width. **Conclusions:** Incorporating platelet indices (PIs) into platelet-rich plasma (PRP) preparation protocols and methodologies is recommended to optimize the recovery of denser, more reactive, and regenerative platelets inherent in peripheral blood, which are integral to the patient’s homeostatic tissue repair mechanisms. Pre-procedural assessment of the patient’s platelet biological status allows for the preservation of an optimal platelet profile within PRP preparations, potentially enhancing clinical efficacy.

## 1. Introduction

In 2020, Everts et al. published a comprehensive review on platelet-rich plasma (PRP), covering preparation methods, platelet dosing, and leukocytic effects in relation to the innate and adaptive immune systems, angiogenesis, and pain modulation [1]. This narrative review, conducted five years later, aims to provide an updated clinical perspective on PRP performance, focusing on a gap in the literature: systemic homeostasis and its impact on platelet volume and density. This review provides insights into this critical area to further the understanding of PRP’s regenerative potential and improve patient outcomes. The human body, via homeostasis, inherently regenerates and repairs tissues through stem and progenitor cells, platelet-derived growth factors, signaling cells, cytokines, and other molecules [2]. Platelets are the first cellular responders following vascular damage or an impairment of tissue integrity. Patients with musculoskeletal (MSK) or spinal disorders and other ailments have an unmet need for innovative and less invasive treatment options. The use of biological and non-surgical treatments to address these maladies is rapidly advancing. One such treatment, PRP, is being extensively studied [3,4]. In orthobiological applications, PRP demonstrates promising results and often meets the goals of regenerative medicine therapies aiming to restore degenerative or defective tissue by establishing new functional tissue. Unfortunately, there are still no definitive and accepted standards for preparing orthobiological bioformulations of PRP [5,6]. Because of this lack of consensus, PRP preparation protocols may not produce a sufficient platelet dose to alter the local microenvironment, leading to variability in outcomes in clinical studies. This lack of consistency in reported outcomes leads to conflicting recommendations for clinical use in specific MSK pathologies [7,8,9,10]. Additionally, in the preparation of PRP products, minimal consideration has been given to the patient’s biological status following the occurrence of homeostatic tissue responses [11]. These are natural physiological responses following tissue injury, chronic inflammation, and immune disorders [12,13]. More specifically, the innate homeostatic repair response of patients leads to megakaryocyte (MK) heterogeneity. Considering that MKs are precursors to platelets, it is inevitable that downstream heterogeneity in platelets would also be observed [14,15]. This heterogeneity in platelet structures, including their size, content, protein expression profiles, and age, has resulted in a great deal of interest in clinical pathologies [16].

This evidence-based narrative review discusses the importance of platelet subpopulations and their accompanying platelet densities, as well as the mean platelet volume (MPV) and platelet distribution width (PDW). MPV levels and platelet concentrations in various PRP fractions are considered in an experimental model following a single-spin and double-spin centrifugation procedure. Additionally, clinical data are presented on changes in MPV, platelet concentrations, and red blood cell (RBC) concentrations following double-spin PRP preparation and relevant statistical associations are analyzed.

This manuscript is presented as a combined original research and narrative review, synthesizing the current literature on platelet heterogeneity, mean platelet volume, density, and homeostatic regulation, and integrating original experimental and clinical analyses that empirically illustrate and support the proposed framework.

## 2. Platelet-Rich Plasma Preparation and Whole Blood Density Separation

PRP devices and their preparation methods have advanced significantly over the past decade, utilizing the density differences in cellular elements in blood for separation with centrifugation. Each PRP production method yields a different characterization of PRP, with variations in platelet concentration, leukocyte and RBC levels, and molecular content, proteins, and extracellular vesicles, complicating the definition of an optimal PRP product, particularly since platelets can have a density similar to leukocytes.

Currently, autologous PRP for MSK use is defined as a concentrated, liquid, cellular fraction of fresh anticoagulated peripheral blood where the platelet count exceeds 1 × 10^6^/μL [7]. At the point of care, PRP specimens can be prepared with either simple low-volume kits or devices for larger volumes, with similar preparation protocols.

The latter devices utilize swing-out, horizontal centrifuges that involve single-spin and double-spin centrifugation protocols. With a double-spin protocol, whole blood cells are generally more concentrated and better separated from other cells, based on their individual cell densities, as shown in Table 1. Platelets are the lightest cells, followed by leukocytes, and RBCs are the densest. Furthermore, platelets have a diameter measuring 2–5 μm, while that of leukocytes is about 20 μm. Notably, in the clinical PRP literature addressing classifications, preparation protocols, and mechanisms of action, platelet density is portrayed as a single fixed value or ranging from 1.040 to 1.065 g/mL (Table 1). These values have been used as references in the literature regarding PRP technology and preparation protocols [17,18,19].

The fundamental characteristic of PRP is the total platelet count available for patient treatment, which depends on the patient’s platelet concentration, the volume of predonated whole blood, and the specifics of the PRP preparation process. Key factors include the number of centrifugation cycles, g value, and duration. While various combinations of these variables are mentioned in the literature, only a few studies provide a systematic comparison and analysis of these factors [20].

### Differences Between Single- and Double-Spin Platelet-Rich Plasma Devices

Depending on the design characteristics of the device, PRP devices can use either single- or double-spin protocols, at the discretion of the end-user. After a first centrifugation spin, whole blood is separated into three fractions. The upper plasma fraction contains platelets and some leukocytes. The second, intermediate, fraction is the buffy coat containing more platelets and leukocytes, while its bottom layer comprises RBCs. The third fraction is the full RBC layer.

For single-spin PRP devices, this concludes the centrifugation cycles and PRP is ready to be extracted in the next preparation step. Notably, the PRP controller now decides on the PRP extraction method and volume and thereby determines the characteristics of the bioformulation of the PRP specimen regarding platelet numbers and the presence or absence of leukocytes. To date, most single-spin PRP products are clinically utilized to avoid any buffy coat cells and demonstrate a clear yellow PRP product, created from the acellular plasma layer, capturing only low-density cells, and therefore largely exclude leukocytes, erythrocytes, and denser platelets. Typically, these devices have a low platelet capture rate and PRP volume [21,22,23].

During a double-spin preparation protocol, the subsequent step involves removing the plasma and buffy coat layers from the erythrocyte layer, followed by a second centrifugation. The operator may include cells originating from the interface between the buffy coat and erythrocyte layer, thereby capturing leukocytes and potentially some denser platelets. Post-centrifugation, cellular components—including platelets—are separated based on their respective densities . After resuspending the isolated cells in a minimal volume of plasma, the practitioner collects the PRP volume as anticipated. Ideally, PRP preparation protocols should be adaptable, allowing clinicians to generate treatment samples with a defined therapeutic platelet concentration and bioformulation tailored to the patient’s specific pathoanatomical lesion.

## 3. PRP Effectiveness in Orthobiological Applications

Platelets are the progeny of MKs. Because they lack nuclei, platelets have often been considered simple cell fragments performing cell-like functions, although their role in hemostasis and thrombosis is widely recognized. The enormous potential of platelet function is well known and has led to many autologous-prepared blood derivates, with an assortment of PRP formulations being used as orthobiologics to treat various MSK and spinal pathologies. Although the plateletcrit (PCT), defined as the total volume or mass of platelets, is low compared to other whole blood cellular constituents [24], platelet transcriptomic materials play regulatory roles and enable the functional diversity of platelets [1,25]. Among other factors, platelets contain a diverse abundance of platelet growth factors, cytokines, and mRNA-encoding proteins and microRNAs [26,27]. In orthobiology, these specialized proteins, molecules, and signaling cells play increasingly important roles in biochemical and molecular processes in the local microenvironment in an array of pathoanatomical tissue structures.

Examples of platelet roles in orthobiological applications include tissue repair and regeneration and the bridging of innate and adaptive immune mechanisms to contribute to immunomodulatory processes [28]. PRP concentrates can contribute to anti-inflammatory processes, initiate angiogenesis, jumpstart wound healing [7,29,30,31], and facilitate antinociceptive effects through their capacity to interact with, or influence, various resident and immune cells and mesenchymal stem cells and platelet interactions with leukocytes [1,7]. However, from a pathophysiological perspective, platelets also contribute to various inflammatory disorders [32], as well as to cancer metastasis [33].

Unfortunately, a consensus on the biological effectiveness of PRP regarding platelet dosing and bioformulations is lacking, although increasing evidence suggests a strong correlation between platelet dosing and positive patient outcomes [4,34,35]. The lack of a dynamic regulation that includes PRP centrifugation methods and validation of PRP specimens to determine the cellular composition and (platelet) concentration is undoubtedly responsible for the variability in the literature, including reported negative patient outcomes [8,9,36,37,38,39]. Furthermore, with more than 35 commercial PRP systems available on the market, each with its own preparation method, the likelihood to achieving consensus on PRP preparation protocols is almost null [22]. Surprisingly, few studies have considered the individual patient’s biological status prior to a PRP application, other than the fact that low platelet concentrations might affect treatment outcomes.

## 4. New Perspectives in Platelet Biology Relevant for PRP Preparations

Recent advances in platelet biology have revealed new insights into platelet formation, function, heterogeneity, signaling, and communication [40]. These enhancements together provide new insights into the various roles of platelets in health and disease and should be considered in the preparation of PRP products as they strengthen the potential for PRP to act as a disease-modifying biological product targeting chronic inflammation, as in osteoarthritis (OA), and induce tissue repair and regeneration. Emerging evidence has suggested that PIs, a group of platelet analytics obtained from an automatic blood analyzer, have diagnostic and prognostic values in certain diseases [24].

Concerning PRP products, the implementation of these new and informative platelet dynamics can optimize platelet concentrations and doses.

### 4.1. Heterogeneity of Megakaryocytes

MKs, large cells that are differentiated from hematopoietic stem cells (HSCs), reside mainly in the adult bone marrow (BM) and are the precursors of platelets. Following megakaryopoiesis, MKs increase their ploidy, size (50–100 µm in diameter), mature, and release proplatelets into the peripheral circulation, where they are converted into platelets [41,42]. Den Dekker et al. described major MK heterogeneities occurring during their HSC differentiation program [43]. Interestingly, BM is recognized as a primary site of platelet production, with indirect evidence suggesting a potential pulmonary role in platelet biogenesis. This process involves the release of platelets from both mature and immature MKs, as well as hematopoietic progenitors, as highlighted by Lefrançais et al. [44].

The existence of distinct MK subpopulations and their functional heterogeneity and ontogenic features with indications of platelet cellular heterogeneity were recently confirmed by Liu et al. [15].

Interestingly, it is thought that chronic tissue inflammation leads to reactive thrombopoiesis prompted by interleukin 6 (IL-6) [45]. As a result, IL-6 stimulates proplatelet formation, resulting in an increase in platelets released from the BM to the peripheral circulation.

### 4.2. Platelet Subpopulations

The MK heterogeneity provides a source of differentiation between platelet subpopulations within individuals, with differences in size, function, life span, and expression of surface receptors being observed in each of these subpopulations [46]. Accordingly, platelet subpopulations have been defined based on these different platelet profiles [47]. Additionally, platelet alpha granules are not a homogeneous population as they consist of distinct subpopulations, each with unique cargo and functions [48]. The ability of platelets to modulate inflammation and participate in immune responses is attributed to their specialized surface receptors and molecular granular content, which exert procoagulant, aggregatory, and secretory functions [49]. Because platelets perform a multitude of functions, it is not surprising that they alter their size and phenotype to fulfill their functional roles [50]. Platelet heterogeneity varies greatly and can modify the transcriptional and translational landscape, based on patients’ pathological circumstances, by exhibiting a predetermined biochemical heterogeneity [51,52,53,54]. The literature indicates that platelet subpopulations and heterogeneity play an important role in immune surveillance and inflammation [49,55].

Platelet age, defined as reticulated or immature and non-reticulated or mature, has been identified as another significant variable involved in platelet subpopulations [56]. Reticulated, freshly released, platelets from MKs appear to have increased RNA content compared with mature platelets, as well as more dense granules and higher levels of platelet surface activation markers [40]. In contrast, older platelets present a decrease in cytoskeletal proteins, lower mitochondria numbers, and granular secretion [57]. Different platelet subpopulations have been recognized to play distinct roles in the prognosis, incidence of adverse events, and mortality of both cardiovascular diseases and infectious diseases such as COVID-19 [58,59,60].

#### Platelet Density Variations

Numerous hematological studies have been performed in healthy individuals to determine the variations in platelet number, age, density, size, and volume. Notably, it has been found that humans have a wide range of platelet densities, ranging from 1.040 to 1.091 g/mL, with equivalent MPV values [61,62,63,64,65]. Strikingly, this broad range is a strong divergence from the platelet densities mentioned and adopted in the PRP application technology and applications. Martin et al. measured platelet density and its correlation with volume and concluded that there is a positive correlation between the MPV and density. Only the densest platelets deviate from this correlation [66]. These research groups have suggested the existence of platelet subpopulations, as a means of simplifying the interpretation of individual platelet densities. Platelet densities have been subdivided into low-, intermediate-, high-, and extreme density fractions. Accordingly, the MPV ranges and the PDW percentages per fraction are presented in Table 2. Therefore, we believe that it is invaluable to consider that patients can have different platelet sizes with unpredictable platelet densities when preparing PRP specimens.

## 5. Platelet Indices

Complete blood count (CBC) analyzers are increasingly being used in orthobiological procedures to validate the PRP injectate as a quality parameter, determine the platelet dose, verify the presence of leukocytes, and establish the effectiveness of a PRP preparation procedure. Additionally, hematology analyzers provide indices related to platelet and erythrocyte counts [67]. For more in-depth platelet analysis, PIs have been introduced. These parameters are derived from hematology analyzers and pertain to the morphology and proliferation of platelets, as well as the number of platelets, reflected as platelet concentrate per unit of measure [68]. PIs are being investigated as potential new biomarkers in the diagnosis and prognosis of various acute and chronic diseases [69]. PIs include the MPV, PDW, platelet/large cell ratio (P-LCR), and the PCT [24]. These novel blood-based platelet parameters are easily accessible, at no additional cost, and may have potential as novel biomarkers of several acute and chronic diseases [70,71]. MPV is the most commonly investigated platelet parameter and is used as a marker of platelet function, reactivity, and activation [72,73].

A fundamental relationship between large, dense, and reactive platelets in the circulation and medical conditions is supported by numerous clinical studies. Elevated MPV levels have been observed when platelets are obtained from patients with pathological processes and conditions such as thrombocytopenia, hypertension, and stroke [74,75,76,77]. Other studies have demonstrated a significant correlation between increased MPV levels and inflammatory diseases [78,79]. Interestingly, several studies have examined the MPV as a potential biomarker of patient prognosis, with most of these studies linking higher MPV values to worse clinical conditions or outcomes [79]. More specifically, in the field of orthobiologics, MPVs are significantly higher in patients with hip OA and synovitis [80].

The PDW represents a distribution curve of platelets measured at the level of 20% relative height in a platelet histogram, with a total curve height of 100%. The PDW is a marker of platelet anisocytosis, namely a large variation in the size of platelets. The PDW describes the size distribution of platelet sizes as they are produced by MKs, and the value increases upon platelet activation [81].

Changes in the MPV and PDW are always in the same direction: if the MPV increases, the PDW also increases, and vice versa [82]. The P-LCR represents the percentage of platelets with MPVs greater than 12 fL in the circulation. A characteristic of this marker is its susceptibility to changes in platelet size [24].

Lastly, the PCT reflects the proportion of pure platelets in whole blood and is derived from the platelet count and the MPV [24].

### The Effect of Exercise on Platelet Indices

Acute strenuous exercise reliably alters both the number and morphology of circulating platelets. Because the spleen sequesters roughly one-third of the body’s total platelet pool, adrenergic stimulation during intense effort mobilizes this reservoir into the bloodstream, producing a well-characterized thrombocytosis [64]. The magnitude is substantial and dose-dependent, with a rise in platelet count of approximately 24% above baseline after strenuous exertion [83]. Morphology shifts in parallel, as MPV rises alongside count by about 3.7% after vigorous activity, around 1.5% after moderate work, and consistently across all resistance intensities. Furthermore, Anz et al. studied the impact of PRP product composition following pre-draw exercises and found that 20 min of vigorous cycling at 70–85% of maximum heart rate increased platelet concentration by >20% in whole blood and significantly in both a plasma-based and a buffy coat-based PRP device [84].

## 6. Homeostatic Tissue Repair Response and Hematopoietic Consequences

In MSK pathologies, when the balance between anabolic and catabolic signals is interrupted, chronic inflammation, pain, tissue degeneration, and loss of function are often observed. Subsequently, this initiates a unique physiological response and repair mechanism, tissue homeostasis. 

### 6.1. Homeostatic Regulation and Communication

Tendon and joint homeostasis are not completely understood. However, the literature indicates that the sensory functions in the nervous system and specific neuronal mediators play an active role in pain regulation, inflammation, and homeostasis [85,86]. This activates a unique response and repair mechanism in numerous specialized cells [13]. In mammalian physiology, homeostasis plays a central role, and many aspects of systemic homeostatic responses are well understood. In systemic, or localized, pathological situations, homeostasis and morphogenesis mechanisms will be initiated in response to this situation [87]. However, homeostasis at pathoanatomical tissue levels and its biological repair mechanisms and consequences are rarely discussed in the orthobiological literature.

Prior to PRP preparations, awareness of a homeostatic tissue repair response and how it might relate to platelet density and MPV will help to optimize platelet dosing and bioformulation strategies, most likely leading to positive patient outcomes.

### 6.2. Neuronal Regulation and Bone Marrow Cells

**Figure 1:**
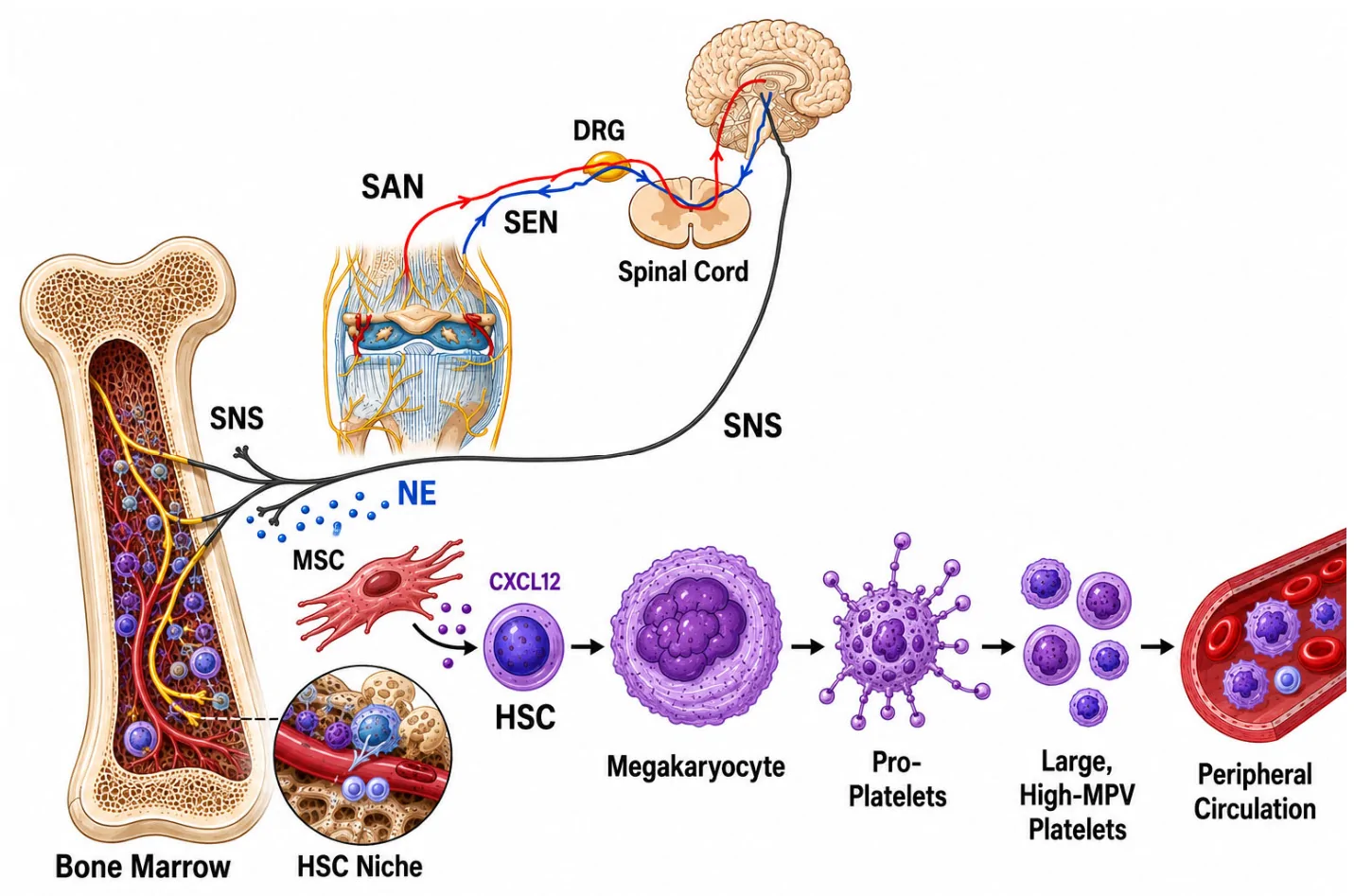
Graphic illustration of the “brain-bone-blood triad” in knee OA. Inflammatory mediators produced by the synovium degrade cartilage and are released as MMPs and ADAMTs. Subsequently, the SAN signals via the DRG to the brain. In response to homeostatic cues, SENs release reparative and anti-inflammatory molecules that inhibit further cartilage degradation. Additionally, the autonomic SNS penetrates deep into the BM, reaching the HSC niches. In this local microenvironment, neuronal signaling regulates hematopoiesis. SNS outflow increases NE release, which acts on MSCs, reducing CXCL12 levels. As a result, lower CXCL12 concentrations promote HSC egress from their niche, inducing hematopoiesis. Large MKs produce proplatelets that release platelets with a large MPV into the circulation. Abbreviations: SAN: sensory afferent nerve, SEN: sensory efferent nerve, DRG: dorsal root ganglia, SNS: sympathetic nervous system, HSC: hematopoietic stem cell, NE: norepinephrine, MSC: mesenchymal stem cell, CXCL12: C-X-C motif chemokine 12, MMP: matrix metalloproteinase, ADAMTs: A disintegrin and metalloproteinase with thrombospondin motifs.

Homeostasis throughout the body is a complex self-regulating process that requires interplay between a pathological local microenvironment and immune and nervous systems and involves many actions [13]. Given its relevance to our review, we will briefly summarize homeostasis, beginning with the role of the nervous system. Sensory nerves, extending from the dorsal root ganglia of the spinal cord, innervate various tissues throughout the body, including bone, joints, and tendons [86]. These nerves generate signals, detect noxious stimuli, interpret pain, and recognize inflammatory cytokines. These relay signals are then processed and integrated into higher brain centers [88]. Sensory nerve fibers innervate cortical and trabecular bone, BM blood vessels, the periosteum, and other intra-bony structures [89,90]. Nerve fiber receptors initiate intracellular signaling, releasing neuropeptide transmitters, which are crucial in bone metabolism [90]. A shell of vascularized and innervated bone and nerves surrounds BM hematopoietic stem and progenitor cells (HSPCs), mesenchymal stem cells, and endothelial progenitor cells [91,92]. Within the various BM niches, stem cells are functionally associated with endothelial, immune, and BM cell populations. HSCs, leukocytes, and neuronal cells are an essential part of the host defense and tissue repair mechanisms and are regulated by neurotransmitter signaling. Interestingly, the nervous system acts as a critical modulator of processes activated in response to bodily responses such as chronic inflammation, stressful conditions, and malignancies [93]. This dynamic regulation, as illustrated in Figure 1, is called the “brain-bone-blood triad” [94,95] and dictates, either directly or indirectly, stem cell behavior by controlling the self-renewal, proliferation, and differentiation of differentiated terminal cells [96]. Neuronal signaling has been observed to trigger more proliferation and differentiation in HSPCs to produce MKs, proplatelets, and ultimately platelets, which are mobilized to the circulation [97,98]. More specifically, crosstalk among BM, HSPCs, and nerves has been described by Wan et al. as indicating how these specialized cells are involved in bone diseases such as OA, osteoporosis, and heterotopic ossifications [99].

## 7. Clinical Data and Observations

Although PIs are being actively investigated as novel diagnostic and prognostic biomarkers across acute and chronic diseases [79], patients’ platelet density, MPV, and PDW are not considered critical determinants of PRP quality in current preparation protocols—despite varying markedly within a single individual. Because platelets with different MPVs distribute across blood fractions by density after centrifugation, we conducted an experimental study and an extensive statistical analysis of clinical whole blood and PRP treatment vials to assess MPV variance, platelet concentration, and other PIs.

### 7.1. Patients and Methods and PRP Processing Techniques

This clinical analysis supporting this review was a retrospective, single-arm, observational study of consecutively treated patients. Data were collected at 2 clinical sites (L.P. and G.F.) between November 2023 and November 2024. All patients presented for PRP treatment of various MSK pathologies and had peripheral blood drawn as part of routine, standard-of-care assessment immediately before treatment. Blood was collected by venipuncture into 3.2% sodium citrate and analyzed within 15–30 min of blood draw and PRP preparation at room temperature on a single hematology analyzer (Beckman Coulter DxH520, Brea, CA, USA) to minimize preanalytical variation in platelet indices. Data from patients were excluded from analysis if they had any of the preanalytical conditions or medications reported by Korniluk et al. [74] to alter the MPV, such as active acute infection, recent thrombotic events, or antiplatelet therapy, so that the measured platelet indices reflected the patients’ underlying musculoskeletal presentation rather than confounding hematologic factors.

#### 7.1.1. Statistical Analysis of Clinical Data

Statistical analyses were conducted using SAS/STAT software (SAS/STAT version 9.4M8 (Maintenance Release 8, January 2023; SAS Institute Inc., Cary, NC, USA). Descriptive statistics are reported as the mean ± standard deviation. Statistically significant differences between groups were determined using independent sample or paired t-tests as appropriate, with a 95% confidence level on each mean principal effect to account for multiple comparisons. All statistical tests were two-tailed and a *p*-value < 0.05 was considered statistically significant. Regression analyses were performed to quantify the relationship between pairs of quantitative variables. Pearson product/moment correlations were calculated to ascertain the parametric measure of a linear relationship between pairs of variables. An adjusted R-squared (adj-R) value was added; this provides a measure of the amount of variation explained by the independent variable.

#### 7.1.2. Analysis of PIs of Platelet-Rich Plasma Preparations

**Figure 2:**
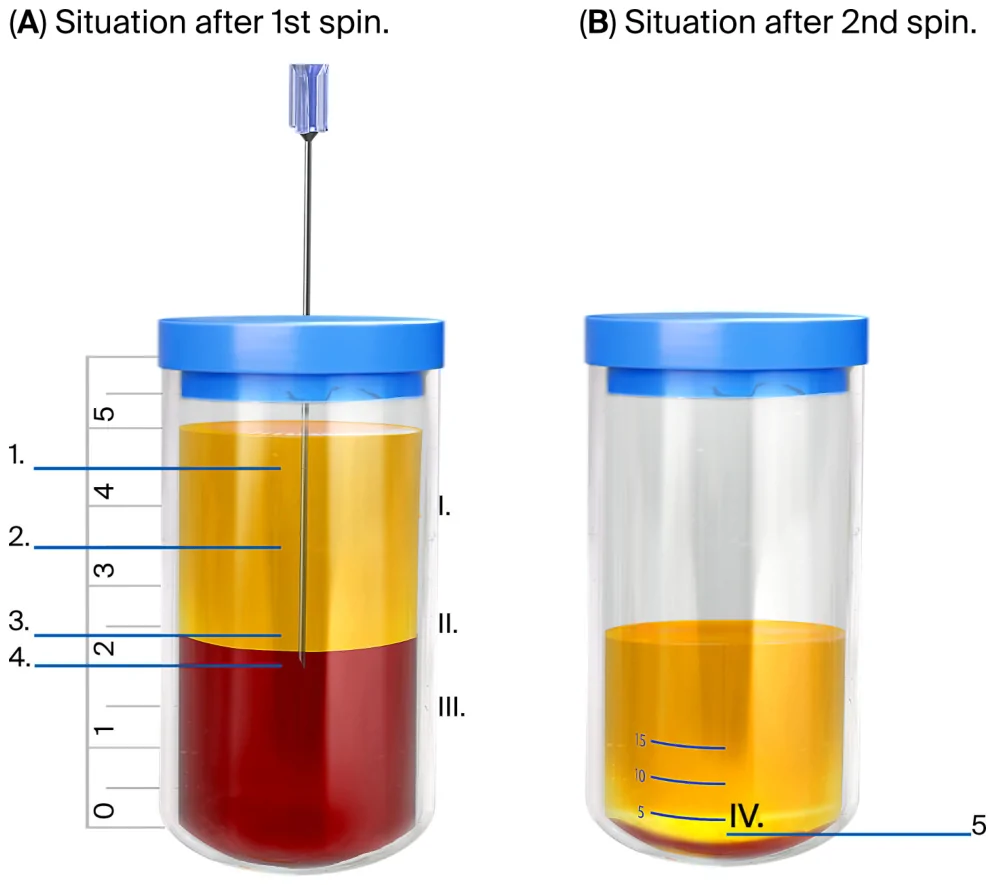
The experimental framework provides a comprehensive, stepwise protocol for fractionating whole blood during PRP preparation and performing targeted sampling across defined layers to evaluate PIs, and CBC parameters. After the initial spin, blood separates into plasma (I), buffy coat (II), and RBC layers based on cellular densities (III). A spinal needle (20 G × 3.5 inches) is inserted centrally at the PRP device’s inlet to withdraw 0.3 mL from each layer at predetermined depths relative to the buffy coat boundary, as marked on a centimeter ruler affixed to the device. Four fractions (1–4) are obtained at specific positions: 0.25 cm below the buffy coat within the RBC fraction (4); directly from the buffy coat (3); 1.25 cm above (2); and 2.25 cm above (1). Following the first spin, these samples are processed to assess compositional differences across the layers. After the second spin, sample (5) is drawn from the final PRP vial (IV) to characterize the final product. To minimize cross-layer interference, sampling from the RBC fraction above the plasma fraction is performed with a 1 mL syringe. The second centrifugation is conducted at 2400 *g* for 5 min, after which additional samples are collected. (Picture courtesy of Dr. L. Podesta).

We hypothesized that, after a unit of whole blood is centrifuged, platelets would be distributed throughout the plasma, buffy coat, and RBC fractions according to their MPV values. To test our hypothesis, we designed an experimental model in which 60 mL of freshly donated anticoagulated blood from ten healthy volunteers who consented (five women and five men, age 52 ± 11 years, not using any medication, free of chronic disease and preanalytical factors that might influence the MPV value, as described by Korniluk et al. [74]) was centrifuged using a single-spin PRP preparation protocol, followed by a double-spin procedure (Figure 2). Before the whole blood specimen was injected into the device, a 0.3 mL anticoagulated whole blood baseline sample was taken for analysis. The first spin—2 min at 2500 relative centrifugal force (RCF), or g-force—produced the classical three-layer separation of whole blood, as shown in Figure 2A. To standardize the PRP device sampling protocol, we identified the buffy coat demarcation line in each device from all volunteers, between the platelet-poor plasma (PPP) and RBC fractions. This line was then used as a reference level for determining all sample positions, using a centimeter ruler that was attached to the PRP device after the first spin. The testing specimen for CBC analysis and MPV determination was therefore always taken from the same level, relative to the buffy coat demarcation line (Table 3).

To evaluate the efficacy of the double-spin PRP procedure and extracted PRP, the PIs were determined in the residual plasma and RBC layers.

### 7.2. PI Parameters in Whole Blood and in Clinical PRP Specimens

Because clinical data regarding PIs following PRP preparations are scarce, we analyzed the hematological data of 389 patients (age 60.1 ± 17.6 years, 226 females, with no previously mentioned chronic diseases) who had been treated for various MSK pathologies by L.P. and G.F. Our objective was to evaluate PI variances in patients’ whole blood compared with PRP treatment specimens. Platelets in PRP should have a similar MPV to those circulating in the peripheral circulation because they are released from MKs in response to homeostasis. Data are shown in Table 4.

#### 7.2.1. Pearson Correlation Analysis

We investigated the statistical associations between the PRP bioformulations administered and the MPV using correlation and regression analysis models. Because RBCs have a higher cell density than platelets, we explored whether denser platelets are trapped in the RBC layer after the second centrifugation spin. Pearson correlation coefficients were calculated to analyze the association between RBCs and MPVs. Data are shown in Table 5. All whole blood and PRP samples were analyzed for CBC and the 5-part differential with the same hematology analyzer (Beckman Coulter DxH520, Brea, CA, USA), with MPV and PDW determination.

#### 7.2.2. Regression Correlation Analysis

Based on the results of the Pearson correlation analysis, which demonstrated a highly significant association between RBCs and MPVs, a regression analysis was conducted and provided further insight into the relationship between RBCs with PRP-MPV as the dependent variable. This analysis of variance revealed a highly significant relationship between RBCs and PRP-MPV (*p* < 0.0001). Our findings are in accordance with the data published by Harrison et al. [20].

### 7.3. Clinical Observations and Implications

The evidence on MPV falls into three distinct tiers that should not be conflated: its diagnostic and prognostic association with disease biology, its value in characterizing PRP product composition, and the more limited evidence linking it to therapeutic outcomes after PRP.

Van Oost et al. unambiguously demonstrated a significant positive correlation among platelet density, the MPV, and the concentration of α-granules in platelets [62]. This was recently confirmed by Pokrovskaya et al., who showed, in a 3D ultrastructural platelet analysis, that the number of α-granules is linearly correlated with platelet size [100]. Consequently, high-density platelets have greater concentrations of multiple platelet growth factors, angiogenesis regulators, and immunomodulatory molecules compared with lower-density platelets [50]. Notably, Davizon-Castillo et al. and Rui et al. observed that newly released large and dense platelets contain more RNA and proteins than mature platelets, indicating a higher platelet exosome presence [101,102]. Comprehensively, younger platelets with higher MPVs have been reported to be metabolically and enzymatically more reactive than smaller platelets [103].

#### 7.3.1. MPV as a Diagnostic and Prognostic Marker of Disease Biology

MPV reflects proinflammatory and prothrombotic conditions during thrombopoiesis, regulated by thrombopoietin and inflammatory cytokines [104], and is associated with obesity, hypercholesterolemia, diabetes, hypertension, cardiovascular disease, and stroke [78]. The direction of change is context-dependent: in rheumatoid arthritis and ankylosing spondylitis, MPV correlated inversely with inflammation, being significantly lower than in controls [105,106], whereas in knee OA with synovitis, MPV was significantly higher than in OA without synovitis (*p* < 0.0001) [78]. Kang et al. similarly linked higher MPV, PDW, and P-LCR to disease activity, inflammatory markers, and bone marrow edema severity [52], with platelet size tracking platelet activity, in accordance with Leader et al. [107]. In hip OA, MPV was highly significantly elevated in severe disease (Kellgren–Lawrence grades 3–4) versus mild–moderate disease (grades 1–2) [80]. Collectively, these findings establish that platelet indices reflect underlying patient biology, but they concern disease characterization rather than PRP quality.

#### 7.3.2. MPV and Related Indices as Descriptors of PRP Product Composition

Beyond their diagnostic value, platelet indices describe what is actually captured in the PRP vial. Our experimental and clinical analyses (Section 7.1 and Section 7.2; Table 3, Table 4, Table 5 and Table 6) show that MPV, PDW, and platelet density distribute across the plasma, buffy coat, and RBC fractions and differ between whole blood and the final PRP product, indicating that preparation method determines which platelet subpopulations are recovered. Because platelet density correlates with α-granule content [61,101], these indices function as measurable descriptors of PRP product composition rather than merely of patient disease state.

#### 7.3.3. Evidence Linking PI’s to Therapeutic Outcomes

The smallest body of evidence bears directly on therapeutic outcome. Uchino et al. related the reticulated-platelet fraction (immature platelet fraction, IPF) in peripheral blood and PRP to VAS scores before and after PRP therapy in knee OA [108], and Kothari et al. found that PRP platelet count significantly affected functional scores and, notably, that MPV correlated with VAS pain scores [109]. These associations are consistent with the hypothesis that higher-quality, denser platelets improve PRP efficacy; however, they remain correlative and hypothesis-generating, and no interventional trial has yet manipulated pre-procedural platelet indices to establish a causal effect on outcome.

### 7.4. Significance of Fluctuating MPVs and Platelet Densities in PRP Specimens

It is generally accepted that PRP platelets contain multiple types of granules. α-Granules constitute the major granule population by size and number and contain platelet growth factors, adhesion factors, and other molecules. Therefore, α-granules are considered significant intra-platelet structures and are key to the efficacy of PRP tissue repair and regeneration. In the literature, many variables have been discussed to optimize platelet capture rates, thereby increasing the PRP platelet concentration and the availability of granules. Frequently cited parameters include the type of anticoagulant, centrifugal acceleration and deceleration, processing time, number of centrifugation steps, PRP extraction method, and monitoring platelet activation. However, individual patient PIs other than platelet concentration, such as the MPV, PDW, and platelet density, are not considered significant fluctuating variables that influence the optimization of PRP treatment specimens. In addition, it is not commonly known that platelets are classified into subpopulations according to their size, MPV, and PDW.

After PRP application with an elevated percentage of dense activated platelets with a high MPV, platelet pathways are significantly activated [1]. In contrast, less dense and older platelets have diminished α-granule secretion upon activation, as measured by P-selectin expression studies in reticulated platelets [57]. PRP specimens comprising less dense platelets with lower MPVs have a lower platelet α-granular content [100] and therefore a reduced regenerative potential. Hence, neglecting the whole blood platelet density, MPV, PDW, and their variabilities prior to PRP preparation will have a significant effect on the quality of platelets and the ultimate platelet capture rate [110,111].

## 8. Discussion

PRP optimization protocols have been examined across a range of clinical settings with the aim of enriching treatment specimens and thereby improving efficacy. Orthobiological procedures should employ PRP technologies that yield specimens containing at least supraphysiological platelet concentrations, that is, ≥1 × 10^9^ platelets/mL [1,112]. Beyond concentration alone, these non-surgical biotherapies require accurate delivery of an adequate platelet dose, and, therefore, of a sufficient payload of platelet-derived growth factors, cytokines, chemokines, and related mediators. Only under these conditions can the physiological cascades governing on-site immunomodulation, inflammatory resolution, and angiogenesis be initiated and tissue repair ultimately achieved [7,105].

A recurrent limitation of the field is the lack of standardization in PRP preparation, which produces substantial biological variability in the platelet component of the final product [6,113]. Such cellular variability may compromise repair processes, most notably angiogenesis, by impairing restoration of an adequate microvascular network. Clinical results after PRP procedures are generally favorable, yet several trials have failed to demonstrate benefit over placebo, and some have reported adverse effects [8,9,36,37].

To date, the patient’s biological status at the time of treatment has neither been discussed nor considered as a determinant of PRP quality. To our knowledge, this is the first report to propose individual patient biological status as a variable that should be assessed and accounted for during PRP preparation. Platelet biogenesis occurs in response to the host homeostatic reaction to pathology, mediated through the brain–bone–blood axis. Under acute or chronic inflammatory conditions, nociceptive and neurotransmitter signaling from the pathoanatomical site reaches the central nervous system, which in turn activates BM sensory nerve fibers innervating intraosseous structures [104]. Through skeletal interoceptive regulation, BM hematopoiesis is triggered, and hematopoietic stem and progenitor cells (HSPCs) are stimulated toward the terminal differentiation of highly specialized MKs [58,93]. Couldwell et al. demonstrated that environmental stressors, including acute and chronic inflammation and other local or systemic pathologies, alter MK differentiation prior to the release of dense, reticulated platelets into the peripheral circulation [106]. Mechanistically, MKs reorganize their cytoskeleton into proplatelets, from which large, dense, reticulated platelets are fragmented and released; proplatelets distribute newly formed platelets along a log-Gaussian size distribution, such that platelets of heterogeneous rather than uniform MPV are generated [114].

In presenting this framework, it is important to delineate which of its constituent elements rest on established mechanisms and which represent the integrative hypothesis advanced here. Several links in the proposed chain are individually well supported. Platelet heterogeneity with respect to size, density, granule content, and age is firmly established [46,47,51], as is the positive correlation among MPV, platelet density, and α-granule cargo [60,66,100]. Neuronal regulation of bone marrow hematopoiesis through the brain–bone–blood axis [95], and the capacity of inflammatory mediators such as IL-6 to drive reactive thrombopoiesis [45], are likewise grounded in experimental hematology. Density-based centrifugation and the differential recovery of platelet subpopulations by single- versus double-spin devices are similarly demonstrable empirically, including in the dataset reported here (Table 3, Table 4, Table 5 and Table 6). What remains conceptual and is offered as a unifying hypothesis rather than a proven pathway, is the proposition that these separately validated phenomena operate as a single continuous axis linking a patient’s homeostatic tissue-repair state to the cellular composition of the PRP injectate and, ultimately, to therapeutic response. The clinical evidence bearing on this final step is at present associative rather than causal: studies such as those of Uchino et al. [108] and Kothari et al. [109] report correlations between the reticulated-platelet fraction, MPV, and symptomatic improvement, but no interventional trial has yet manipulated pre-procedural platelet biological status and prospectively measured its effect on outcome. We therefore frame the homeostatic blueprint as a mechanistically plausible model that assembles established biology into testable predictions—principally, that PRP enriched in high-MPV, high-density platelets will outperform conventional low-density preparations—while explicitly acknowledging that this causal inference awaits prospective validation.

Platelets with a higher MPV are correspondingly larger and denser, and, as reported by Kuter, denser platelets are metabolically more reactive and contain a greater number of α-granules than smaller, less dense platelets [103].

The geometry and physical characteristics of PRP devices, the associated centrifugation parameters, and the fidelity with which preparation protocols are executed jointly determine the quality and bioformulation of the final PRP vial [115]. Centrifugation remains the most widely used method for density-based separation of whole blood: centrifugal and gravitational forces redistribute blood within the device, with differences in cell size and density driving separation [116]. Piao et al. recently developed a comprehensive theoretical model of whole-blood centrifugation for PRP preparation to predict maximum platelet and leukocyte capture rates [117]. Although the authors described the determinants of platelet capture in detail, including platelet density and the customization of centrifugal conditions, they treated platelet density as a fixed value of 1.050 g/mL and thus did not accommodate variation in platelet volume and, consequently, density. This assumption has direct practical consequences. Single-spin devices combined with protocols that deliberately exclude leukocytes and red blood cells (RBCs), whose densities overlap with those of high-MPV platelets, necessarily yield specimens depleted of large, young, and more reactive platelets. Optimized single-spin procedures that control spin time and relative centrifugal force (RCF), and that involve precise aspiration of a defined plasma volume above the buffy coat together with an equivalent volume of buffy coat, can achieve platelet capture rates of up to 72% [118]; centrifugation times beyond approximately 5 min confer no additional benefit [20]. In routine practice, however, most single-spin devices process a limited whole blood volume, and operators frequently exclude leukocytes and RBCs to obtain a clear “yellow” PRP preparation, thereby discarding the larger platelets that sediment with them. Low-volume single-spin devices therefore exhibit reduced platelet capture and retain a substantial platelet fraction within the device after extraction [22,119,120]. Emerging evidence nonetheless indicates that at least one single-spin system can generate distinct bioformulations with complete buffy coat capture and a statistically significant increase in PRP MPV relative to baseline [121].

Double-spin protocols apply predetermined g-forces and durations to generate a stratified buffy coat in which cellular components are ordered by density. Such devices are generally reported to produce superior preparations, because they establish an organized multicellular buffy coat and achieve higher platelet capture rates than low-volume single-spin devices [116]. Importantly, double-spin systems process the entire plasma fraction obtained after the first spin, including platelets across the full MPV range, and flexible protocols permit additional buffy coat and/or RBC material to be incorporated into the final processing step to produce concentrates of defined cellular composition. These methodological differences underlie the profound dissimilarities in PRP characteristics reported in the literature, chief among them the differential ability to capture high-MPV platelets.

Consistent with this, an animal study comparing three centrifugation protocols on low-volume, single-spin devices configured to produce leukocyte-poor PRP while excluding leukocytes and RBCs found that MPV was significantly higher in whole blood than in either the PRP or the PPP fraction, with no differences among protocols [122]. Platelet concentrations, MPVs, RBCs, and leukocyte counts were determined. After processing, the MPV was significantly higher in the whole blood than in the PRP and PPP fractions, with no differences among the different centrifugation protocols.

Preparation methods should therefore be capable of capturing and concentrating high-MPV platelets from a unit of whole blood, because platelet density correlates closely with α-granule content [62]. Biswas et al. reported a significant positive correlation between vascular endothelial growth factor (VEGF) concentration and MPV, with concordant platelet distribution width (PDW) values [123]. This is of relevance given the central role of VEGF in the angiogenic cascade underlying repair of orthobiological pathologies. Where a device cannot capture and concentrate high-density peripheral blood platelets, as is characteristic of low-volume single-spin systems, VEGF, other platelet-derived growth factors, and cytokines are correspondingly reduced in the treatment specimen. It is reasonable to infer that the absence of adequate quantities of these granular mediators will delay or preclude re-establishment of angiogenesis, as recently proposed by our group [7].

In a clinically informative study, Uchino et al. quantified the release of reticulated platelets, expressed as the IPF %, following reactive platelet hematopoiesis in patients with knee osteoarthritis (OA [108]. High whole blood IPF correlated significantly with improvement in VAS scores, whereas whole blood platelet concentration correlated negatively with VAS improvement, implicating reticulated platelets specifically rather than total platelet number. The authors proposed that a high circulating fraction of dense, reticulated platelets provides a favorable substrate for the preparation of a high-quality PRP injectate. Notably, however, no significant correlation was found between the administered PRP and VAS improvement. PRP (5 mL) was prepared using single centrifugation in a 22 mL tube-type device containing a separation gel. From the reported whole blood and PRP data, the platelet capture rate was 37%, indicating poor capture and a correspondingly low platelet concentration. Furthermore, both IPF (2.0% vs. 1.6%) and MPV (10.6 fL vs. 10.1 fL) were higher in the whole blood than in the PRP, confirming that the preparation did not retain the high-MPV platelets present in the circulation. Four considerations follow: first, low-volume devices constrain the absolute number of platelets available for concentration; second, after a single spin, a large platelet fraction remains in the PPP, as evidenced by the 37% capture rate; third, the number of platelets trapped within the gel separator during processing is unknown; and fourth, material below the gel separator is physically inaccessible, so denser and more reticulated platelets are irretrievably retained within or beneath the separator, in the buffy coat fraction.

Because platelets distribute across multiple PRP fractions as a function of their heterogeneity (Table 3), the traditional assumption of a largely constant platelet density during PRP preparation requires revision. This is supported by several studies, including research on OA, reporting significantly elevated MPV and PDW in response to the homeostatic mechanisms that physiologically regulate tissue repair. Recognizing platelets as intrinsically heterogeneous with respect to density and MPV and developing the technical capacity to capture the more reactive subpopulations would yield bioformulations substantially enriched in α-granule constituents, which are fundamental to immunomodulation, angiogenesis, and tissue repair [48]. Conversely, simplified PRP technologies and their associated protocols capture predominantly low-density, low-MPV platelets with reduced α-granule content, and the resulting products possess correspondingly lower regenerative potential. Experimental work comparing four centrifugation methods [124] and the clinical findings of Uchino et al. [108] both indicate that homeostatic responses enrich the peripheral circulation with higher-quality, more reactive platelets that these devices fail to recover.

Several comparative evaluations of PRP devices have been published, focusing principally on differences in platelet counts and other cellular components [21,22,125]. These studies consistently distinguish single-spin from double-spin methods, with double-spin devices achieving higher platelet capture rates and relatively greater clinical efficacy. None, however, addressed the importance of MPV. Ideally, devices and their processing techniques should avoid discarding platelets from the plasma and buffy coat fractions, rather than selectively capturing low-density platelets while excluding high-MPV platelets [110]. Table 3 shows higher MPV in PRP produced using two-spin protocols than in whole blood (8.4 fL vs. 7.55 fL), indicating efficient capture of dense platelets. After PRP extraction, both the residual plasma and RBC fractions contained minimal dense platelets, consistent with high platelet recovery from the device (Table 6). We further identified a highly significant correlation between PRP MPV and RBCs, indicating that larger platelets have cellular densities comparable to those of RBCs; after the second spin, denser platelets were located deeper within the RBC stratum (*p* < 0.001). A significant Pearson correlation between high-MPV platelets and PRP RBCs was demonstrated.

A limitation of our interpretation warrants explicit acknowledgment. MPV and platelet density are correlated but dimensionally distinct indices and are not interchangeable. MPV quantifies the mean of the platelet volume distribution, an intensive, morphometric property of individual cells, whereas density denotes platelet concentration per unit volume, an extensive property of the population [107]. Although larger platelets are younger, richer in granules, and more pro-thrombotically reactive [126], size and number are governed by partially independent mechanisms (megakaryocyte ploidy, thrombopoietic drive, splenic pooling). Elevated MPV thus neither entails nor predicts elevated concentration, and the two are frequently inversely related [125]. The functional superiority of large platelets is moreover agonist- and assay-dependent rather than uniform [50], and MPV is further confounded by preanalytical variables independent of the true population [127]. MPV and platelet concentration should therefore be interpreted as orthogonal, complementary parameters rather than substitutes for one another.

Further clinical studies are warranted and should incorporate patient biological status as an explicit variable, given the expected differences in platelet heterogeneity, MPV, and platelet density. Such studies should characterize the associations between PIs and patient outcomes after PRP biotherapy. Including MPV and PDW as reported parameters would additionally allow practitioners to discriminate between the quality of PRP specimens prepared using different devices, and thereby to improve clinical efficacy and patient outcomes.

## 9. Conclusions

PRP and other non-surgical orthobiological therapies have demonstrated efficacy across a spectrum of musculoskeletal disorders, with numerous reports of favorable patient outcomes. Nonetheless, the literature also documents inconsistent results, influenced by heterogeneous preparation protocols, lack of consensus on PRP classification, and variability in platelet dosing and bioformulations across devices. The patient’s biological status following homeostatic responses is frequently overlooked as a critical factor. Many studies on standardization and outcomes did not incorporate platelet heterogeneity, MPV, or platelet density, despite these parameters directly impacting PRP quality. Regardless of device or PRP extraction technique, precise targeting of plasma and buffy coat fractions is essential for capturing higher-density platelets. This principle applies to any buffy coat-based separation method. To maximize orthobiological PRP efficacy, refined, adaptable preparation methods that integrate individual homeostatic and hematological status should be developed to optimize PRP applications.

## Figures and Tables

**Table 1:** Blood component densities classified into three different groups, as reported by different authors. A: Araki et al. [19]; B: Alves et al. [18]; C: Miron et al. [17].

Blood Component	Density-A (g/mL)	Density-B (g/mL)	Density-C (g/mL)
Plasma	1.025	1.026	
Platelets	1.032	1.058	1.040–1.065
Monocytes	1.063–1.085	1.062	1.055–1.085
Lymphocytes		1.070	
Neutrophils		1.082	
Erythrocytes	1.032	1.100	1.095–1.100

**Table 2:** Overview of platelet subpopulations. Platelet density ranges (g/mL) are categorized in 4 groups, each with their own corresponding MPV range in percentages. Table is modified from published data, as referenced in the table. Abbreviations: MPV, mean platelet volume; PLT, platelet concentration.

Platelet Density Range	MPV Range (%)	PLT Density Range (g/mL)
Low	6.01 ± 0.39	1.040–1.065 [62,63,64]
Intermediate	7.37 ± 0.28	1.065–1.070 [62,64]
High	8.21 ± 0.56	1.070–1.080 [62,64]
Extreme	>9	1.080–1.091 [61]

**Table 3:** PIs and RBC values of baseline whole blood, four fractions after the first spin, and after the final PRP preparation from 10 healthy volunteers. Data are presented as mean values ± standard deviation. *p*-values represent the statistical change from the previous fraction. * *p* is the statistical result of the comparison of the baseline values with the final PRP specimen. All values were statistically significant, except the *p*-values in italics. Abbreviations: PI: platelet indices; BL, baseline; RBC, red blood cell; PRP, platelet-rich plasma fraction; MPV, mean platelet volume; PDW, platelet distribution width; PLT, platelet concentration.

Parameters	BL	Fraction 1.	Fraction 2.	Fraction 3.	RBC 4.	PRP 5.
		After 1st spin				After 2nd spin
MPV (fL)	7.08 ± 0.51	7.16 ± 0.38	7.19 ± 0.32 *p* = 0.034	7.62 ± 0.36 *p* < 0.0001	8.39 ± 0.21	7.38 ± 0.19 *p* = 0.0325, * *p* = 0.066
PDW (%)	18.9 ± 1.6	18.6 ± 0.4	19.7 ± 0.6 *p* = 0.005	19.5 ± 0.3 *p* = 0.402	18.1 ± 3.3	19.9 ± 0.4 *p* = 0.0431, * *p* = 0.248
PLT (×10^3^/µL)	234 ± 51	461 ± 72	519 ± 80 *p* = 0.008	652 ± 122 *p* = 0.005	49 ± 19	2152 ± 535 *p* = 0.0007, * *p* < 0.0001
RBC (×10^6^/µL)	4.32 ± 0.28	0.0 ± 0	0.00 ± 0 *p* = 0.109	0.77 ± 0.18 *p* = 0.0008	7.54 ± 0.66	1.49 ± 1.15 *p* = 0.162, * *p* = 0.002

**Table 4:** PIs and RBC values of baseline whole blood and PRP specimens following clinical PRP analysis in 389 patients. The results are mean values ± standard deviation and minimum and maximum values. Abbreviations: PI: platelet indices; SD, standard deviation; PRP, platelet-rich plasma fraction; MPV, mean platelet volume; PDW, platelet distribution width; RBC, red blood cell concentration.

	Whole Blood Mean ± SD	Min; Max	PRP Mean ± SD	Min; Max	*p*-Value
MPV (fL)	7.55 ± 0.67	6.08; 14.9	8.4 ± 0.81	6.34; 12.08	*p* < 0.0001
PDW (%)	15.0 ± 2.6	11.5; 35.8	19.7 ± 0.9	18.0; 24.41	*p* < 0.0001
PLT (×10^3^/µL)	196 ± 51	74; 387	2082 ± 639	512; 3.999	*p* < 0.0001
RBC (×10^6^/µL)	3.80 ± 0.5	2.1; 7.8	1.3 ± 1.5	0.01; 5.54	*p* < 0.0001

**Table 5:** Relationships between the RBC fraction in the PRP, MPV, and PDW. Pearson correlation coefficients were calculated using a two-tailed test to determine the *p*-value.

	PRP-RBC	PRP-MPV	PRP-PDW
PRP-RBC	1	0.50082	0.56632
		*p* < 0.0001	*p* < 0.0001
PRP-MPV	0.50082	1	0.6345
	*p* < 0.0001		*p* < 0.0001
PRP-PDW	0.56632	0.6345	1
	*p* < 0.0001	*p* < 0.0001	

**Table 6:** PIs of residual plasma and RBC fractions after PRP extraction. Data are presented as mean values ± standard deviation. *p*-values represent the statistical difference between the PRP fraction and the PPP and RBC fractions, indicating the efficacy of the double-centrifugation protocol. Abbreviations: PI: platelet indices; PPP-F: platelet poor plasma fraction; RBC-F, red blood cell fraction; PRP, platelet-rich plasma; MPV, mean platelet volume; PDW, platelet distribution width; PLT, platelet concentration.

Platelet Indices	PPP-F	RBC-F	*p*-Value—PRP
MPV (fL)	7.83 ± 0.44	7.903 ± 1.01	*p* < 0.0001
PDW (%)	8.9 ± 0.6	19.13 ± 6.7	*p* < 0.0001
PLT (×10^3^/µL)	18 ± 8	32 ± 10	*p* < 0.0001

## Data Availability

No data are available due to privacy restrictions.

## Abbreviations

The following abbreviations are used in this manuscript:

ADAMTs	A Disintegrin and Metalloproteinase with Thrombospondin Motifs
BC	Buffy Coat
BL	Baseline
BM	Bone Marrow
CBC	Complete Blood Count
CXCL12	C-X-C motif Chemokine 12
DRG	Dorsal Root Ganglia
HSC	Hematopoietic Stem Cell
HSPCs	Hematopoietic Stem and Progenitor Cells
IL	Interleukin
IPF	Immature Platelet Fraction
KL	Kellgren–Lawrence
LP	Lower Plasma Fraction
MK	Megakaryocyte
MMP	Metalloproteinase
MPV	Mean Platelet Volume
MSC	Mesenchymal Stem Cell
MSK	Musculoskeletal
NE	Norepinephrine
OA	Osteoarthritis
P	*p*-value
PCT	Platelet Crit
P-F	Platelet Fraction
P-LCR	Platelet Large Cell Ratio
PLT	Platelet
PDW	Platelet Distribution Width
PRP	Platelet-Rich Plasma
RBC	Red Blood Cell
RCF	Relative Centrifugal Force
SAN	Sensory Afferent Nerve
SEN	Sensory Efferent Nerve
SNS	Sympathetic Nerve System
UP	Upper Plasma Fraction
VAS	Visual Analog Scale
